# Undue burden: Black faculty, COVID-19, and the racial justice movement

**DOI:** 10.1017/cts.2022.460

**Published:** 2022-09-13

**Authors:** Tracy M. Layne, Uraina S. Clark, Nihal E. Mohamed, Sarah J. Miller, Jamilia R. Sly, Holden E. Kata, Varuna Astha, Steven A. Lawrence, Yvette Hutson, Kirk N. Campbell, Emma K.T. Benn

**Affiliations:** 1 Blavatnik Family Women’s Health Research Institute, Icahn School of Medicine at Mount Sinai, New York, NY, USA; 2 Department of Population Health Science and Policy, Icahn School of Medicine at Mount Sinai, New York, NY, USA; 3 Department of Obstetrics, Gynecology, and Reproductive Science, Icahn School of Medicine at Mount Sinai, New York, NY, USA; 4 Center for Scientific Diversity, Icahn School of Medicine at Mount Sinai, New York, NY, USA; 5 Department of Neurology, Icahn School of Medicine at Mount Sinai, New York, NY, USA; 6 Department of Urology, Icahn School of Medicine at Mount Sinai, New York, NY, USA; 7 Grossman School of Medicine, New York University, New York, NY, USA; 8 Division of Nephrology, Department of Medicine, Icahn School of Medicine at Mount Sinai, New York, NY, USA

**Keywords:** Minority tax, diversity, equity, inclusion, racial justice, health inequities, workforce diversity, time and effort tax

## Abstract

A crucial reckoning was initiated when the COVID-19 pandemic began to expose and intensify long-standing racial/ethnic health inequities, all while various sectors of society pursued racial justice reform. As a result, there has been a contextual shift towards broader recognition of systemic racism, and not race, as the shared foundational driver of both societal maladies. This confluence of issues is of particular relevance to Black populations disproportionately affected by the pandemic and racial injustice. In response, institutions have initiated diversity, equity, and inclusion (DEI) efforts as a way forward. This article considers how the dual pandemic climate of COVID-19-related health inequities and the racial justice movement could exacerbate the “time and effort tax” on Black faculty to engage in DEI efforts in academia and biomedicine. We discuss the impact of this “tax” on career advancement and well-being, and introduce an operational framework for considering the interconnected influence of systemic racism, the dual pandemics, and DEI work on the experience of Black faculty. If not meaningfully addressed, the “time and effort tax” could contribute to Black and other underrepresented minority faculty leaving academia and biomedicine – consequently, the very diversity, equity, and inclusion work meant to increase representation could decrease it.

## Dual Pandemic Climate

The year 2020 saw the collision of two global pandemics: the coronavirus disease 2019 (COVID-19) and racial injustice. In the USA, the COVID-19 pandemic is described as a syndemic where pre-existing conditions (e.g., diabetes and cardiovascular disease) and social factors (e.g., fragmented healthcare) interact to create excess morbidity and mortality in marginalized groups[1] (i.e., those in the numerical and sociopolitical minority) [2]. Marginalized groups, inclusive of underrepresented racial/ethnic groups (URGs) such as Black populations, have higher rates of the chronic conditions that increase the risk of COVID-19-related morbidity and mortality relative to White populations and occupy a disproportionate number of the essential worker roles that increased their susceptibility to the virus [3–5].

Until recently, inequities experienced by the Black community were not broadly considered in the context of systemic racism [6] – a foundational component of health and justice inequities [7]. This delay in meaningful acknowledgment of the impact of systemic racism occurs despite health disparities described in the Institutes of Medicine’s “Unequal Treatment” in 2003 [8], and similar articulations by public health and social science scholars who have been championing this work for decades [9,10,11]. The recent contextual shift to recognize racism as a root cause [12] occurred as the health and economic devastation of the pandemic played out alongside the continued senseless murder of Black people captured on video. Specifically, the Black Lives Matter (BLM) movement, a contemporary extension of past movements defending the humanity of Black people [13], became mainstream after a captive global audience created by the pandemic quarantine watched police murder George P. Floyd, Jr. on May 25, 2020. The BLM movement subsequently motivated demands for accountability for police misconduct and broader global calls for racial justice reform [14], including in academic and biomedical spaces [14–17]. In fact, leading clinical, academic, and biomedical entities have begun to acknowledge the relationship between systemic racism and the inequities experienced by Black professionals in their respective fields. Described as a synemic diversity, equity, and inclusion (DEI) initiatives are a common approach to remedying such disparities [18]. Given the overlapping disproportionate impact of the COVID-19 pandemic and the concurrent pandemic of racial injustice [6] on Black communities [7], we focus our examination of their dual impact on Black faculty (e.g., in teaching, research, and/or clinical roles) in academia and biomedicine through the lens of DEI work.

URG faculty and staff in academic, biomedical, and other settings often confront an undue burden, referred to as the “minority tax,” disproportionately placed on them to participate in institutional DEI work [19–21]. Participation in these efforts includes a combination of recruiting, mentoring, teaching, committee membership, and conducting trainings or workshops designed to, directly or indirectly meet the institution’s stated DEI goals. A distinctive consequence of these activities [22] is they often hinder the academic advancement of URGs [21], effectually “taxing” groups for whom there are significant career achievement gaps (e.g., research grant funding, publications, and promotion) when compared to their White counterparts [23]. This tax is a well-described challenge [19,20] brought to the forefront of our collective consciousness, along with the other long-standing societal maladies of racial and ethnic health inequities and systemic racism, by the dual pandemics of COVID-19 and racial injustice. Here, we discuss the same phenomenon, however, instead use the term “time and effort tax” given the current movement toward language and descriptors that avoid further stigmatizing marginalized groups [24]. While we, a predominantly Black group of scholars in biomedicine, focus on Black faculty, there is no question that the challenges described herein cut across marginalized groups (e.g., as described by race/ethnicity, ability, immigration status, and LGBTQIA + identity), especially for those whose identities are intersectional.

Acknowledgement of systemic racism (and not race) as the central driver of health and justice inequities defines much of the current dual pandemic climate and inspired the institutional statements and initiatives designed to address it. As this work unfolds, we consider how this climate could intensify the time and effort tax on Black faculty and present an operational framework that can be used to inform rigorous scientific enquiry and recommendations in this regard.

## Pressure on Career Advancement

As with other classic examples of the “tax,” the engagement of Black faculty in institutional efforts to support equity through DEI can have the unintended consequence of overextending those who are often either one of the few or the only URG in their department and/or institution [14,20]. As documented in the pre-COVID-19 era, assignment of DEI work, which is not typically counted in the overall assessment of success [20], often belies the very concept of “protected time” for research and therein jeopardizes the career advancement of those URG faculty who are overrepresented in these efforts. At the height of the dual pandemic, there was a simultaneous emphasis on COVID-19-related funding opportunities (more money for research) and antiracist initiatives (non-research). Navigating this landscape of research opportunities and DEI responsibilities can create a consequential imbalance for Black faculty in independent investigator positions responsible for securing funding for their work and maintaining their research productivity [25]. This is especially true for those at institutions that may be under financial strain due to pandemic-related revenue losses, limiting the resources available to support Black researchers who have taken on DEI roles. Support in this context, whether it be staff, technical resources, and/or funding, is vital given well-documented racial/ethnic achievement gaps in research [23,26–28]. Overall, there are significant disparities in federal funding for research among underrepresented groups [23,27], with Black and Asian female investigators less likely to receive National Institutes of Health (NIH) R01 funding compared to White female investigators in the US [26], a significant marker of success and requirement for promotion at some academic institutions [25]. Black faculty tasked with taking on multiple or a single high-demand DEI role have less time to pursue potentially career advancing opportunities.

Additionally, an increasingly competitive funding environment for health disparities research (HDR) is important given findings that the Black-White funding gap in the US is linked, in part, to the tendency of Black investigators to focus on public health-related research targeting disease prevention and intervention (e.g., socioeconomics, inequities, lifestyle), which receive lower rates of NIH funding [23]. Yet, data indicate that even among applications focused on these topic areas, Black investigators are less likely to be funded compared to their White counterparts [23]. Considering the smaller relative number of Black faculty in academia and biomedicine, the institutional pressures for them to participate as change agents [14], and the existing funding landscape, Black investigators are increasingly vulnerable to a time and effort tax often associated with DEI efforts, which consequentially may continue the pattern of disparate research funding.

When the pandemic is eventually over, and the attention and interest in antiracism likely wanes, health inequities and racial justice will still matter and their collective impact will still weigh heavily on Black faculty. Recommendations to address the tax on career advancement in this context include protection of time for early career faculty [22] re-evaluating the meaning of research excellence [14], and institutions demonstrating the value they place on DEI through investment in and compensation of those doing the DEI work [14,29].

## Commitment vs. Burden

Many institutions have made antiracism statements and/or begun to prioritize health equity and antiracism in response to the dual pandemics. Without comprehensive examination of these efforts and changing structural policies and practices, these efforts are thought to be more effective at masking rather than eliminating systemic racism [25]. Many Black faculty feel a passion and commitment to be active in the movement towards eliminating systemic racism [14,20]. Those whose research address issues that disproportionately impact marginalized communities – their communities – can also feel a tremendous burden to champion these DEI efforts in the professional spaces they occupy [21]. Notably, Black faculty are both more likely to be engaged in HDR [23] and to lead institutional DEI efforts. Both feelings of commitment and burden can be amplified in the dual pandemic climate.

For example, Black faculty in DEI roles may feel helpless or struggle when confronting the expectations for transformative change from younger generations. Moreover, the change in perspective that occurs as Black faculty gain positions of power but continue to confront significant barriers to fundamental change can translate into a repeating cycle of helplessness over sequential generations. This experience is further complicated if appointed to DEI roles without the structural support, compensation, or protected time to facilitate success of the work [18]. Underlying much of this is the burden of assumptions that any given individual Black faculty member can, or wants to, serve as the spokesperson [30] or representative of the lived experiences of an entire racial/ethnic group [7]. Research in this area suggests the individual and collective weight of these challenges negatively shapes the experiences of Black faculty in academia [7,20,21,30]. The time and effort tax places an undue burden on Black faculty to address problems that affect them and the institution at large without the shared responsibility for change or improvement [20]. Understandably, recommendations put forth to address this undue burden include expanding responsibility to all members of an institution, [12,29,31] but also providing financial support and intangible support in the form of time, expertise, and mentorship of those in DEI leadership and advocacy roles [29].

## Well-being: a Physical and Emotional Toll

Black faculty are not immune to the dual pandemics impact on their communities; the consequences are present in their personal and professional lives. In this heightened moment, there is more attention to the perspective of Black faculty describing the ways in which their lived experiences with this “tax” [32] and racial bias [7,33] impact them both emotionally and professionally. In a commentary, Dr Sophie Balzora, M.D. describes her experience with the tax and the dual role of being a Black female in academic medicine [32]. Dr Balzora explains her expectation of being “othered” and tokenized by those in her White male-dominated field because *“….intersectional discrimination born from individual, institutional, and systemic racism and sexism is pervasive and embedded in the culture of medicine*” [32]. Being othered, tokenized, and stereotyped has been expressed among Black medical students [33], clinicians, and scientists [7]. Professional status does not provide insulation from health inequities and racial injustice.

We see further evidence of this with the death of Dr Susan Moore, M.D., a Black family physician who died of COVID-19 after she documented, via a viral video, what she described as racist treatment [34]. As noted in an op-ed, her death is an example of the “*injustice and intersection of being a healthcare provider and a person of color during COVID-19*” [34]. Despite her professional background, Dr Moore’s advocacy for her own healthcare and humanity did not save her life. Her death is yet another illustration that other people’s perception of Black racial identity, regardless of how a Black person may experience it, can be life-threatening [14]. Conversely, an individual’s assessment of their own racial identity in terms of how the broader society views (or values) Black people, be it positively or negatively [35], also shapes their experiences. Both external and internal perceptions of Black racial identity are consequential, and in the context of observing those that look like you affected by the pandemic and/or racial injustice, knowing someone who experienced it, or experiencing it yourself, the impact can be profoundly negative. The time and effort tax adds another layer of complexity as Black faculty are tapped to address DEI goals without addressing their proximity to the devastation caused by the pandemic and racial injustice.

The current racial justice movement requires that uncomfortable conversations take place in order to promote antiracist environments. Consequently, Black faculty may experience the stress of increasing microaggressions [14,21] – derogatory or negative slights that, whether intentional or unintentional, can be psychologically harmful [30]. Microaggressions were found to be commonly reported among female minority medical students [36], and associated with negative psychosocial well-being, self-doubt, and isolation in the classroom among Black students [37]. In response to these and other uncomfortable interactions, Black faculty may go through the emotionally taxing process of censoring themselves in the expression of their views on or experiences with racial injustice out of fear of being perceived as “angry,” a dreaded stereotype that leaves little to no room to be rightfully angered by injustice. In addition, there are concerns that truly speaking truth to power in demanding the fundamental changes needed to achieve the diversity, equity, and inclusion espoused by institutions, Black faculty run the risk of retaliation and/or social isolation, and potentially compromising their careers [14]. Recommended approaches to mitigate these stressors include the creation of peer support and affinity groups and “tax advising” where trusted senior colleagues can help Black and other URGs strategically navigate the demands of DEI work [22].

## Dual Pandemic Climate and DEI Work: An Operational Framework for Intervention

**Fig. 1.**
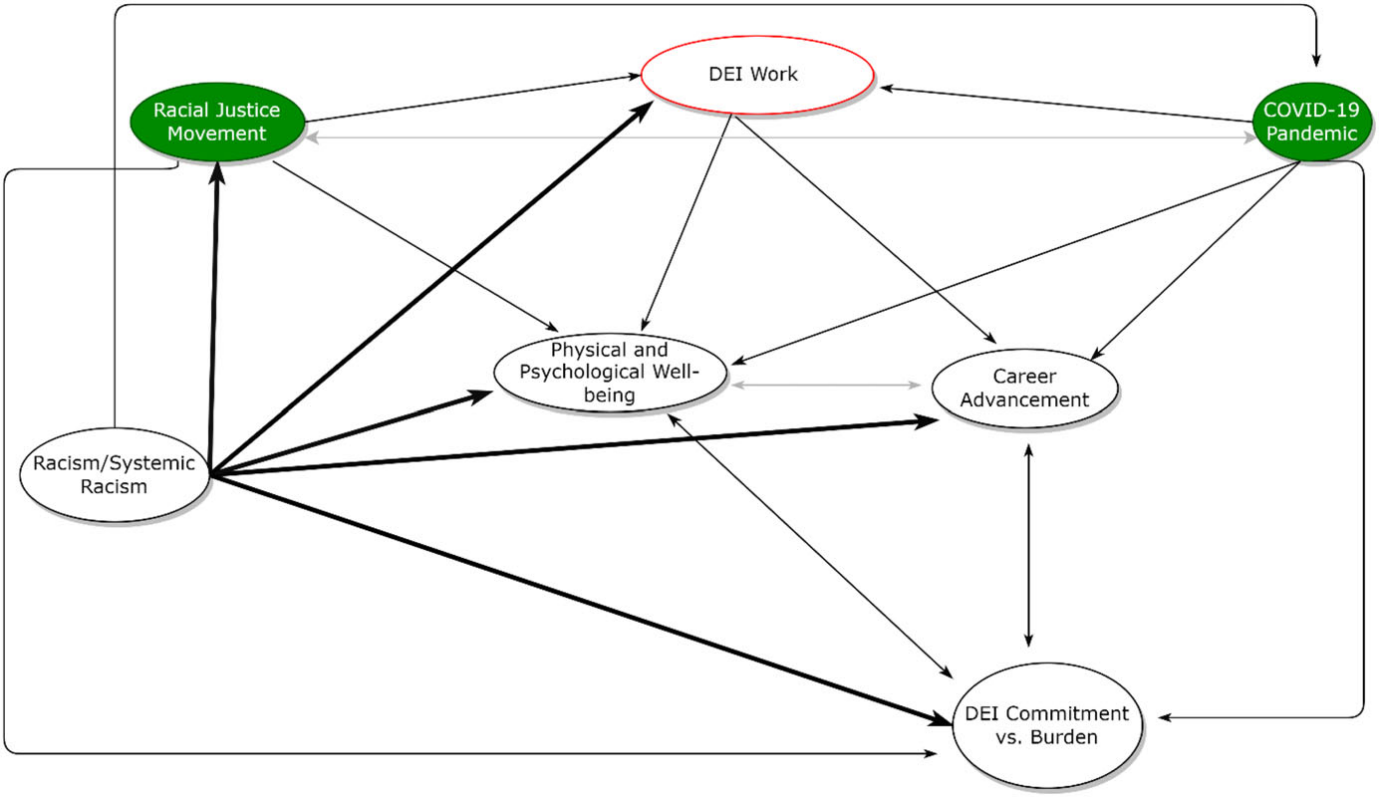
An operational framework for the interconnected relationship between the dual pandemics, diversity, equity, and inclusion (DEI) work, and the career advancement, sense of commitment to DEI, and well-being of Black faculty in academia and biomedicine. The green filled ovals indicate each of the dual pandemics. Bolded arrows indicate pre-pandemic relationships stemming from systemic racism. Non-bolded arrows indicate the additional relationships stemming from the dual pandemics.

The COVID-19 pandemic and the racial justice movement have jointly motivated DEI work that is necessary, but also potentially harmful when the targets of systemic racism are expected to be the primary agents of change against it. Above, we describe the ways in which Black faculty’s well-being, sense of commitment to DEI, and ultimately, their career advancement, are jeopardized in the dual pandemic climate. Recommendations to address these challenges exist, including those that call for directly confronting the underlying racism/systemic behind them. Related to this, we believe one way to support direct approaches to fundamental change in this regard is to ground DEI work in rigorous conceptual frameworks. Figure 1 depicts one such framework from which to begin thinking both broadly and specifically about the ways in which to avoid this “tax” in the dual pandemic climate and beyond.

This dynamic framework, which should evolve over time, is intended to spark consideration of the ways in which each framework pathway could guide the design, implementation, and evaluation of change-oriented policies. It can be used to inform scholarly research and interventions aimed at hypothesis generation, program development, advocacy, and recommendations in ways that leverage the choice and agency institutions have to redress current and/or past dynamics underlying the “tax.” No one pathway in this framework is a panacea for addressing systemic racism in this context. However, any one of the outlined pathways can have a multilevel impact due to the unfortunate pervasive and interconnected relationships between systemic racism, the dual pandemics, DEI work, and the experience of Black faculty.

## A Crucial Reckoning for Academia and Biomedicine

The heightened need for DEI work in the dual pandemic climate requires that academic and biomedical institutions doing this work, who are at the forefront of providing care, conducting research, and developing treatments and vaccines for COVID-19, take care to ensure equity in the distribution of DEI tasks to avoid “taxing” marginalized groups, specifically Black faculty disproportionately impacted by both crises [14]. Rigorous research is needed to quantify the magnitude of this dual burden and to identify efficacious targets for intervention, and we provide a framework as a potential starting point for all groups – inclusive of non-BIPOC groups –- to conceptualize this work.

The time and effort tax highlights one of the ways in which Black people may be relied upon to do the heavy lifting to help move society beyond its systemic racist past and present – through advocacy, leadership, protest, voting, etc. – while many are coping (or not) by living and working through their collective and at times personal trauma. Importantly, the reliance on Black faculty is happening in the context of the constant diminishing of the humanity of Black people in their everyday lives, as is evidenced by the repeated lack of justice for the most egregious acts of violence against them. The lived experiences of Black faculty, in all of its varied forms, shape how they both process the collision of issues at this time and leverage this time to create change. The work at the institutional (and societal) level in support of the BLM movement includes recognition that it is essential that Black and other URG voices are amplified, and that their perspectives form the foundation for DEI efforts. That said, Black people (and other URGs) are not a monolith and cannot be primarily or solely responsible for undoing the racism that targets them. Institutions cannot continue to leverage the voices, experiences, and work of marginalized groups without considering the ways in which overtasking them in these roles can compromise their career advancement and well-being. The unrealistic and unjust expectations associated with the time and effort tax are not sustainable and may contribute to Black and other URG faculty leaving academia and biomedicine. If the “tax” is not meaningfully addressed, the very diversity, equity, and inclusion work meant to increase representation could decrease it.

## References

[1] Mendenhalla E. The COVID-19 syndemic is not global: context matters.The Lancet 2020; 396(10264): 1731. doi: 10.1016/S0140-6736(20)32218-2 PMC7581415 33256917

[2] Helms JE. 4. Toward a methodology for measuring and assessing racial as distinguished from ethnic identity. Multicultural Assessment in Counseling and Clinical Psychology 1996; 8: 143–192. (https://digitalcommons.unl.edu/burosbookmulticultural/8)

[3] Webb Hooper M , Nápoles AM , Pérez-Stable EJ. COVID-19 and racial/ethnic disparities. JAMA 2020; 323(24): 2466–2467. 32391864 10.1001/jama.2020.8598 PMC9310097

[4] Gross CP , Essien UR , Pasha S , Gross JR , Wang S-y , Nunez-Smith M. Racial and ethnic disparities in population level COVID-19 mortality. *medRxiv.* 2020: 2020.2005.2007.20094250. 10.1007/s11606-020-06081-w PMC7402388 32754782

[5] Carethers JM. Insights into disparities observed with COVID-19. Journal of Internal Medicine 2020; 289(4): 463–473. 33164230 10.1111/joim.13199 PMC9325576

[6] Tolchin B , Hull SC , Kraschel K. Triage and justice in an unjust pandemic: ethical allocation of scarce medical resources in the setting of racial and socioeconomic disparities. Journal of Medical Ethics 2020, Online ahead of print. 10.1136/medethics-2020-106457 33067315

[7] Clark US , Hurd YL. Addressing racism and disparities in the biomedical sciences. Nature Human Behaviour 2020; 4(8): 774–777. 10.1038/s41562-020-0917-7 32651473

[8] Smedly BD , Stith AY , Nelson AR. Unequal Treatment: Confronting Racial and Ethnic Disparities in Health Care, 2003. 25032386

[9] Ford CL , Airhihenbuwa CO. Critical race theory, race equity, and public health: toward antiracism praxis. American Journal of Public Health 2010; 100: S30–S35. 20147679 10.2105/AJPH.2009.171058 PMC2837428

[10] Jones CP. Toward the science and practice of anti-racism: launching a national campaign against racism. Ethnicity & Disease 2018; 28(Suppl 1): 231–234. 30116091 10.18865/ed.28.S1.231 PMC6092166

[11] Williams DR , Sternthal M. Understanding racial-ethnic disparities in health: sociological contributions. Journal of Health and Social Behavior 2010; 51(1_suppl): S15–S27. 20943580 10.1177/0022146510383838 PMC3468327

[12] Bailey ZD , Feldman JM , Bassett MT. How structural racism works — racist policies as a root cause of U.S. racial health inequities. New England Journal of Medicine 2020; 384: 768–773. 33326717 10.1056/NEJMms2025396 PMC11393777

[13] Nelson A. The longue durée of Black Lives Matter. American Journal of Public Health 2016; 106(10): 1734–1737. 27626337 10.2105/AJPH.2016.303422 PMC5024410

[14] Obasi AI. Equity in excellence or just another tax on Black skin? The Lancet 2020; 396(10252): 651–653. 10.1016/S0140-6736(20)31536-1 PMC7377687 32711686

[15] Collins FS , Adams AB , Aklin C , et al. Affirming NIH’s commitment to addressing structural racism in the biomedical research enterprise. Cell 2021; 10(12): 3075–3079. 10.1016/j.cell.2021.05.014 34115967

[16] Fontanarosa PB , Flanagin A , Ayanian JZ , et al. Equity and the JAMA network. JAMA Cardiology 2021. 10.1001/jamacardio.2021.2527 34081093

[17] AMA’s Organizational Strategic Plan to Embed Racial Justice and Advance Health Equity, 2021–2023, 2021.

[18] Jordan A , Shim RS , Rodriguez CI , et al. Psychiatry diversity leadership in academic medicine: guidelines for success. American Journal of Psychiatry 2021; 178(3): 224–228. 33641375 10.1176/appi.ajp.2020.20091371

[19] Campbell KM , Hudson BD , Tumin D. Releasing the net to promote minority faculty success in academic medicine. Journal of Racial and Ethnic Health Disparities 2020; 7(2): 202–206. 31953638 10.1007/s40615-020-00703-z

[20] Rodríguez JE , Campbell KM , Pololi LH. Addressing disparities in academic medicine: what of the minority tax? BMC Medical Education 2015; 15: 6. 25638211 10.1186/s12909-015-0290-9 PMC4331175

[21] Gewin V. The time tax put on scientists of colour. Nature 2020; 583(7816): 479–481. 32647354 10.1038/d41586-020-01920-6

[22] Faucett EA , Brenner MJ , Thompson DM , Flanary VA. Tackling the minority tax: a roadmap to redistributing engagement in diversity, equity, and inclusion initiatives. Otolaryngology–Head and Neck Surgery 2022; 166(6): 1174–1181. 35380882 10.1177/01945998221091696

[23] Hoppe TA , Litovitz A , Willis KA , et al. Topic choice contributes to the lower rate of NIH awards to African-American/Black scientists. Sci Adv 2019; 5(10): eaaw7238. 31633016 10.1126/sciadv.aaw7238 PMC6785250

[24] National Association of Hispanic Journalists (NAHJ) asks newsrooms to drop the use of “minority” when referencing communities of color, 2020. (https://nahj.org/2020/08/04/nahj-asks-newsrooms-to-drop-the-use-of-minority/#:)

[25] Stevens KR , Masters KS , Imoukhuede PI , et al. Fund Black scientists. Cell 2021; 184(3): 561–565. 33503447 10.1016/j.cell.2021.01.011

[26] Ginther DK , Kahn S , Schaffer WT. Gender, race/ethnicity, and National Institutes of Health R01 research awards: is there evidence of a double bind for women of color? Acad Med 2016; 91(8): 1098–1107. 27306969 10.1097/ACM.0000000000001278 PMC4965301

[27] Adeliane A , Kalinga C , Asani F , et al. Knowledge is power - an open letter to UKRI. Research Professional News 2022. (https://www.researchprofessionalnews.com/rr-news-uk-views-of-the-uk-2020-8-knowledge-is-power-an-open-letter-to-ukri/)

[28] Detailed ethnicity analysis of funding applicants and awardees 2015–16 and 2019–20. UK Research and Innovation, 2021.

[29] Foster KE , Johnson CN , Carvajal DN , et al. Dear White people. The Annals of Family Medicine 2021; 19(1): 66. 33431395 10.1370/afm.2634 PMC7800738

[30] Chisholm LP , Jackson KR , Davidson HA , Churchwell AL , Fleming AE , Drolet BC. Evaluation of racial microaggressions experienced during medical school training and the effect on medical student education and burnout: a validation study. Journal of The National Medical Association 2020. 10.1016/j.jnma.2020.11.009 33358632

[31] Chen CL , Gold GJ , Cannesson M , Lucero JM. Calling out aversive racism in academic medicine. New England Journal of Medicine 2021; 385(27): 2499–2501. 34951752 10.1056/NEJMp2112913 PMC11014457

[32] Balzora S. When the minority tax is doubled: being Black and female in academic medicine. Nature Reviews Gastroenterology & Hepatology 2020; 18: 1. 10.1038/s41575-020-00369-2 32963339

[33] Ellis J , Otugo O , Landry A , Landry A. Interviewed while Black. New England Journal of Medicine 2020; 383(25): 2401–2404. 33176078 10.1056/NEJMp2023999

[34] Maybank A , Jones CP , Blackstock U , Crear-Perry J. Say her name: Dr. Susan Moore. 2020. (https://www.washingtonpost.com/opinions/2020/12/26/say-her-name-dr-susan-moore/)

[35] Sellers RM , Smith MA , Shelton JN , Rowley SAJ , Chavous TM. Multidimensional model of racial identity: a reconceptualization of African American racial identity. Personality and Social Psychology Review 1998; 2(1): 18–39. 15647149 10.1207/s15327957pspr0201_2

[36] Espaillat A , Panna DK , Goede DL , Gurka MJ , Novak MA , Zaidi Z. An exploratory study on microaggressions in medical school: what are they and why should we care? Perspectives on Medical Education 2019; 8(3): 143–151. 31161479 10.1007/s40037-019-0516-3 PMC6565651

[37] Solorzano D , Ceja M , Yosso T. Critical race theory, racial microaggressions, and campus racial climate: the experiences of African American college students. Journal of Negro Education 2000; 69: 60–73.

